# A Quality-Centered Analysis of Eye Tracking Data in Foveated Rendering

**DOI:** 10.16910/jemr.10.5.2

**Published:** 2017-09-28

**Authors:** Thorsten Roth, Martin Weier, André Hinkenjann, Yongmin Li, Philipp Slusallek

**Affiliations:** Bonn-Rhein-Sieg, University of Applied Sciences, Germany; Brunel University London,, UK; Saarland UniversityGermany; Intel Visual Computing Institute,; German Research Center for Artificial Intelligence (DFKI)Germany

**Keywords:** Rendering, Ray tracing, data analysis, perceived quality, eye tracking, foveated rendering, eye movement, region of interest, gaze

## Abstract

This work presents the analysis of data recorded by an eye tracking device in the course of evaluating a foveated rendering approach for head-mounted displays (HMDs). Foveated rendering methods adapt the image synthesis process to the user’s gaze and exploiting the human visual system’s limitations to increase rendering performance. Especially, foveated rendering has great potential when certain requirements have to be fulfilled, like low-latency rendering to cope with high display refresh rates. This is crucial for virtual reality (VR), as a high level of immersion, which can only be achieved with high rendering performance and also helps to reduce nausea, is an important factor in this field. We put things in context by first providing basic information about our rendering system, followed by a description of the user study and the collected data. This data stems from fixation tasks that subjects had to perform while being shown fly-through sequences of virtual scenes on an HMD. These fixation tasks consisted of a combination of various scenes and fixation modes. Besides static fixation targets, moving targets on randomized paths as well as a free focus mode were tested. Using this data, we estimate the precision of the utilized eye tracker and analyze the participants’ accuracy in focusing the displayed fixation targets. Here, we also take a look at eccentricity-dependent quality ratings. Comparing this information with the users’ quality ratings given for the displayed sequences then reveals an interesting connection between fixation modes, fixation accuracy and quality ratings.

## Introduction

Virtual reality has the major goal of presenting a virtual world in a way that resembles reality as close as possible. Recently, head-mounted displays (HMDs) are becoming widely available, providing a suitable display technology for this purpose. To enable a visually pleasant experience without disturbing aliasing artifacts and visible pixel grids, high display resolutions are required. Early HMDs like the Forte VFX 3D (1997, 263 480 2 = 0.25 million pixels) worked at very low resolutions, while modern HMDs like the StarVR (2016, 2560 1440 2 = 7.37 million pixels) have made a huge step forward in this regard. However, the full retinal resolution including a user’s full dynamic field of view (200° horizontally, 150° vertically) would potentially require a resolution of 32k 24k = 768 million pixels [ 1 ]. Such resolutions are neither achievable by current display technology nor are they tractable by current GPUs. In addition, frames need to be displayed with a low latency and at high frame rates to meet the requirements of the display devices and at the same time reduce nausea caused by a perceptual mismatch of the self-induced motion and the visual response (simulator sickness) [ 2 ]. With the ongoing improvements of pixel densities in HMDs and the current inability to render at the required resolutions while maintaining performance, developing new rendering methods to tackle these challenges is urgently required.

Fortunately, the human visual system (HVS) has several limitations which imply that it is not necessary to provide the highest level of detail over the entire visual field. There is a drop of the eye’s visual acuity with increasing *eccentricities*, where the eccentricity describes the angular deviation from the central optical axis. Thus, one possible approach is to adopt techniques that adjust rendering quality based on the exploitation of a user’s current viewing direction. This process is referred to as *foveated rendering*.

The visual field can be divided in *central* and *peripheral* vision. We go with the definition in [ 3 ], where central vision is defined to include the following areas of the fovea (up to 5.2° from the optical axis), the parafovea (up to 9°) and the perifovea (up to 17°). Larger eccentricities are defined to belong to peripheral vision.

The tracking-based adaptation of rendering quality based on the HVS’ drop in visual acuity requires highly accurate and low-latency eye tracking to determine the point of regard (PoR), i.e., the screen space position currently focused by the user. Moreover, several models of human attention have been used in computer graphics to get a notion of the screen space position without using an active tracking mechanism. Both approaches benefit from insights regarding eye tracking data acquired in a foveated rendering system.

Eye tracking as a way to actively measure the user’s gaze directly has been used by the computer graphics community in various disciplines. A survey on rendering techniques can be found in [ 4, 5 ], while perception-driven geometric processing and mesh simplification are described in [ 6 ]. Another field that uses eye tracking is computational displays, where a survey on various techniques can be found in [ 7 ].

Early work in the field of gaze-contingent and foveated rendering techniques utilized focus assumptions [ 8 ] and visual attention models [ 9, 10 ] instead of employing eye tracking devices. Those early systems su ered from the lacking hardware capabilities for both accurate and low-latency eye tracking as well as computational power to synthesize high-quality images. One of the earliest approaches to speed up rendering using perceptual methods is described in [ 11 ], where eye tracking is used to adapt the sampling frequency on the image plane and in object space in accordance with the spatial acuity of the HVS. Another early system can be found in [ 12 ], adapting the geometric quality of a three-dimensional mesh using an anisotropic simplification system. Most notably this is one of the first publications where binocular eye tracking is used inside an HMD.

However, the eye tracking and graphics hardware was still lacking the necessary accuracy, latency and rendering performance to meet perceptual requirements. Hence, early systems focused primarily on a theoretical analysis, e.g., the general influence of the quality degradation on visual performance, especially on search performance. Watson et al. [ 13 ] demonstrated that image resolution could be reduced by half for peripheral vision without a significant influence on search time. Duchowski et al. [ 14 ] demonstrated that color precision can be reduced for peripheral vision, though not as readily as resolution.

As we take a closer look at the eye tracking data one can ask how precise eye tracking devices need to be in order to be suitable for foveated rendering. Loschky et al. [ 15, 16 ] showed that the update must be started at 5 ms to 60 ms after an eye movement for an image change to go undetected. However, an acceptable delay highly depends on the task of the application and the stimulus size and positioning in the visual field. Ways to measure latency and a discussion on di erent tasks can be found in the work by [ 17 ] and [ 18 ].

Synthesizing images from a 3D scene description is made possible by some basic methods in the field of computer graphics, the most important being rasterization and raybased approaches (ray tracing). A comprehensive description of recent work in the field of perception-driven accelerated and foveated rendering can be found in [ 5 ].

Our analysis is based on the fully adaptive foveated ray tracing technique suggested in [ 19 ]. In addition to the system’s fully adaptive sampling, improvements of temporal stability and a reduction of artifacts in the visual periphery are achieved by incorporating a reprojection technique to improve image quality and fill gaps in sparsely sampled images.

The main idea here is that samples are cached in image space, reprojected to a new view and used to aid the image quality of subsequent frames. In this regard the system most closely relates to temporal anti-aliasing [ 20 ] and the mathematical considerations on how to combine samples temporally [ 21 ]. The unique characteristics of our system come from combining a performance-focused reprojection method based on a coarse geometry approximation with foveated rendering methods, which enables us to generate visually pleasant results at high update rates. The user’s gaze is measured using a binocular SMI eye tracker built into an Oculus Rift DK2 and then used to parameterize the rendering process. The evaluation of our rendering system has shown that the subjective perceived quality is very similar to full ray tracing with benchmarks showing a clearly superior rendering performance.

This paper serves the purpose of extending the short analysis of eye tracking data from our user study which is provided in [ 22 ]. The main objective of this extension is to give better, more detailed insights into the recorded tracking data and a more extensive discussion of the results and the connections between gaze data and subjective perceived quality.

In order to provide context, we describe the basic design of the user study and the recorded eye tracking data. Based on this data we describe how the recorded data is analyzed. Amongst others, this analysis revealed an interesting relation between fixation accuracy and quality ratings for di erent fixation modes.

Although there has been related work on analyzing eye tracking data, there is no work in the context of foveated rendering that thoroughly investigates the link between the subjective perceived image quality, the eye tracking precision and the induced effects when asking users to focus or fixate a target in the image. Tracking precision is measured by the distance between the recorded PoR and the actual location that people were instructed to fixate on predefined paths on a screen. [ 23 ] represent this precision by the standard deviation of these measurements to evaluate their low-cost eye tracking system. [ 24 ] use the glint in the eye to derive a PoR. However, they evaluate the precision in terms of determining the right image quadrant. Although the book by Duchowski [25, ch. 12] contains various strategies to evaluate eye tracking data, the author focuses on di erent aspects like dwell time, saccade detection and denoising.

The main contributions of our work are: 1. An estimation of the tracking precision of an HMDmounted eye tracking device, supported by the evaluation of eccentricity-based quality ratings. 2. An analysis of fixation accuracy based on the data recorded during a quality-focused user study carried out for our foveated rendering system. 3. An analysis of the connection between subjective perceived quality and fixation accuracy, providing possible evidence of the presence of visual tunneling effects and the magnitude of their influence on the user’s perception.

The results are discussed and conclusions are drawn in the according sections at the end of this article, together with some suggestions on how to benefit from our findings in practical systems and how to further improve the suggested methods.

## Methods

### Rendering process

**Figure 1:**
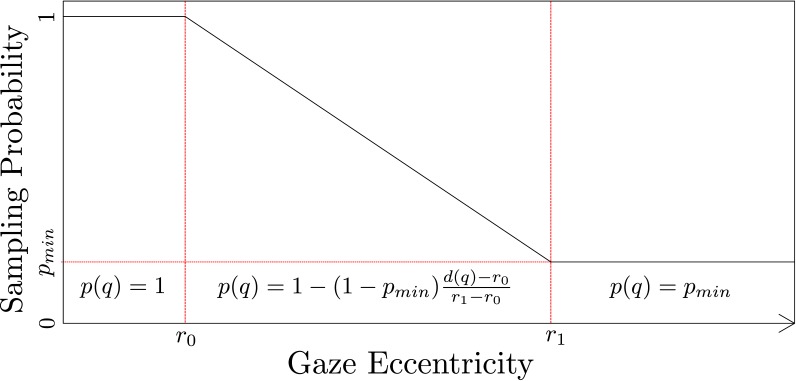
Figure 1. The sampling probability of each individual pixel is computed by evaluating the foveal function with freely adjustable parameters (r₀ ; r₁ ; p_min_). Image adapted from [ 22 ].

As opposed to basic rasterization, ray-based approaches enable us to sample the image plane in a fully adaptive way. This is done by sampling each individual pixel with a probability computed from its eccentricity, based on the foveal function, a fallo function that can be freely parameterized based on current gaze properties and performance requirements. The receptor density of cones in the human eye is approximated quite well with a hyperbolic fallo , which also corresponds to the fallo in visual acuity with increasing eccentricities. Rods, on the other hand, exhibit a density fallo that is much more linear [ 26 ]. In addition to that, visual acuity can also be represented quite well by a linear model when it comes to small angles [ 27 ]. Because of the human visual system’s high sensitivity to peripheral flickering and motion that results from these receptor distributions, we designed the foveal function, to be piecewise linear instead of adopting a hyperbolic falloff , as shown in Figure 1

During each rendering iteration, a set of pixels to be sampled is determined by evaluating the foveal function for each pixel individually. As this results in a sparsely sampled image with only little pixel information towards outer image regions, it becomes necessary to provide a reconstruction method for filling in unsampled image regions. In order to do this, we rely on a reprojection-based approach. 

The support image and support G-buffer provide a low resolution version of the color and geometry information that is computed for each rendering iteration. Based on these, a coarse geometric approximation of the scene geometry as seen from the user’s current point of view is generated, which is then textured with the known color information from the preceding frame. This mesh is then reprojected by rendering it from the camera position of the current frame. The support image is now used to improve areas where reprojection errors due to disocclusions or movement and areas with insu cient quality become apparent. Additional samples are computed where necessary, which is done by analyzing the reprojected image for depth and luminance discontinuities between neighboring pixels. If such discontinuities are found, pixels are scheduled for resampling.

### Evaluation

A specific parameter set for the foveal function is referred to as the foveal region configuration (FRC). An FRC is a triplet (r₀ ; r₁ ; p_min_) that describes the sampling density fallo (cf. Figure 1).

Rendering performance (and thus speedups) compared to full ray tracing depend strongly on the chosen FRC. Our user study has shown good results for the subjective perceived image quality for the medium-sized FRC (specific parameters are shown below). The speedups achieved in our test scenes with this FRC ranged from 1.46 to 4.18 depending on the quality settings (with better speedups for higher quality rendering). The benchmarks were run on an Intel Core i7-3820 CPU with 64GiB of RAM and an NVIDIA GeForce Titan X graphics card at a resolution of 1182x1464 pixels. The chosen FRCs are based on the necessity of achieving a frame rate that has to be at least as high as the display refresh rate of the HMD utilized in our user study. Thus, while it may be chosen as large as possible within the range that still provides the required performance, it is also desirable to leave some room for additional computations such as physics. Our user study gives clues about the possible parameter range for FRC adjustments.

**Figure 2:**
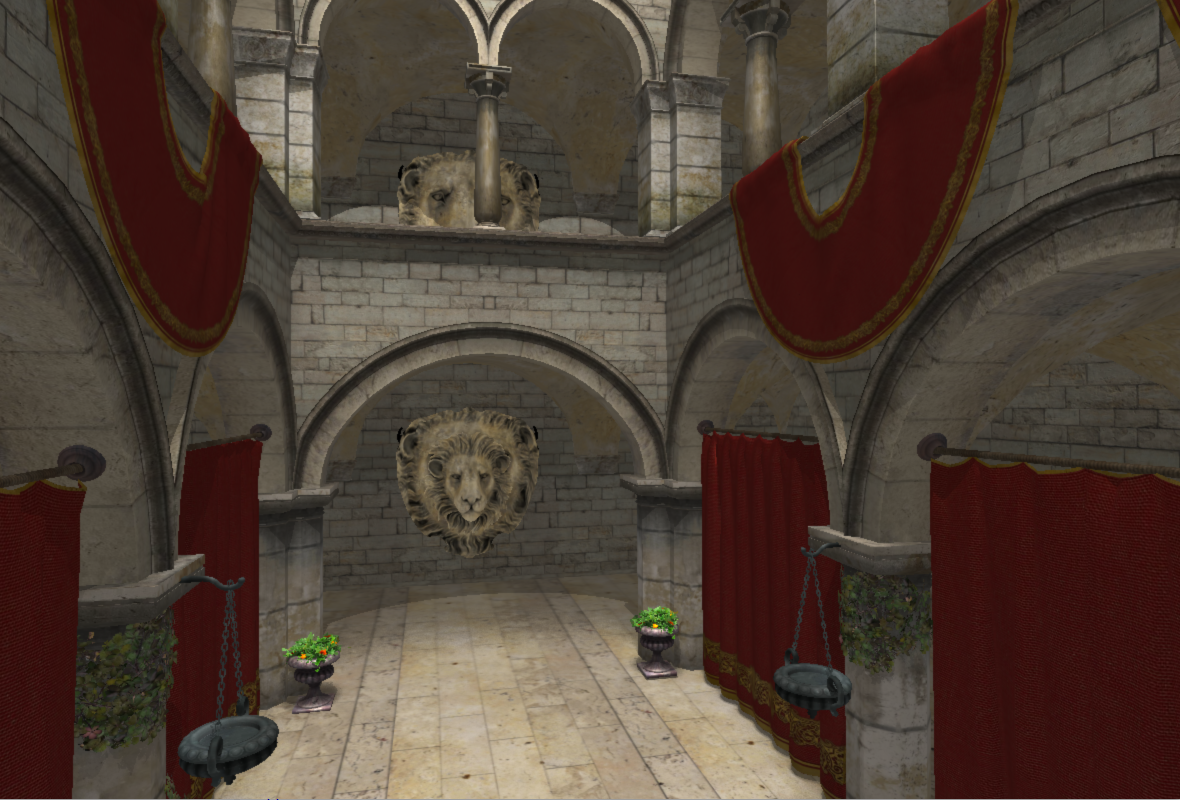

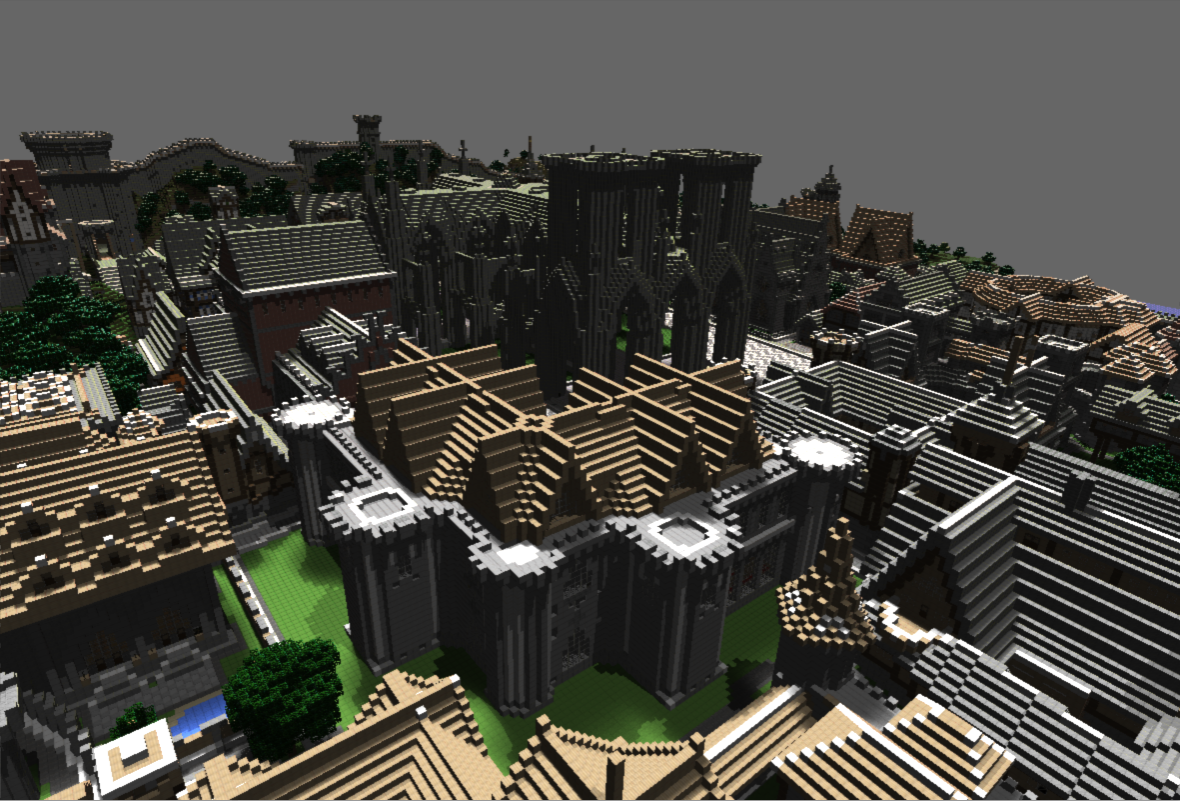

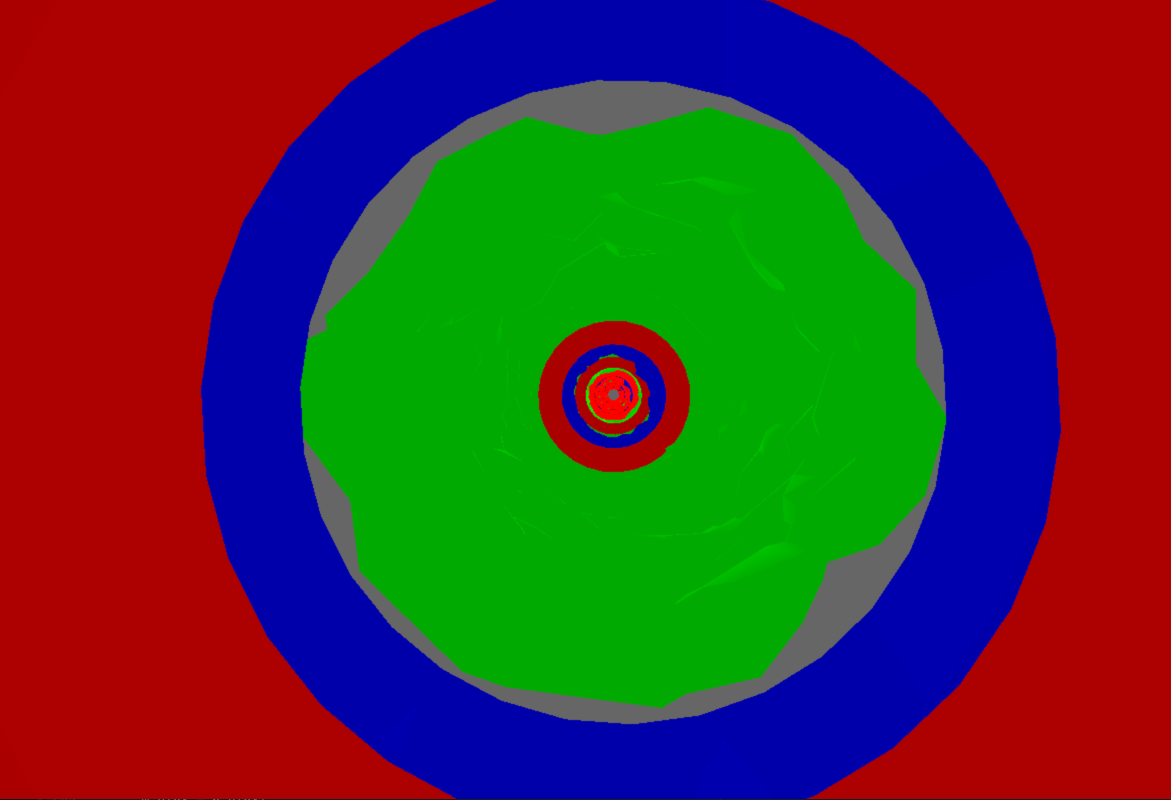

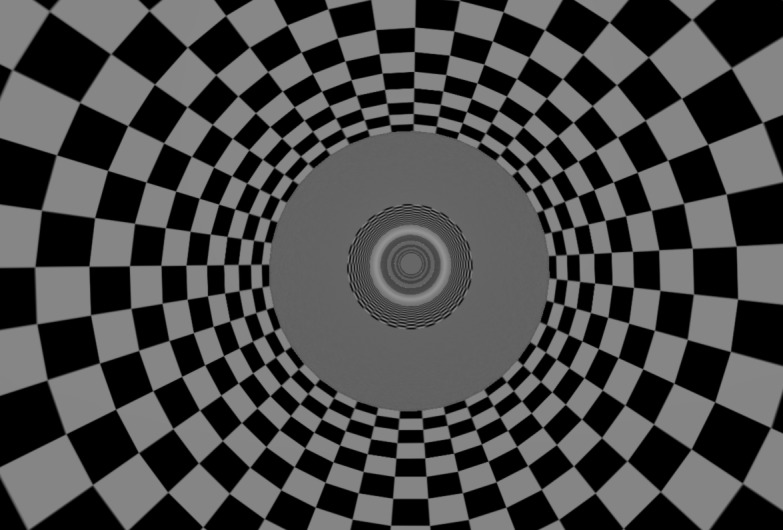

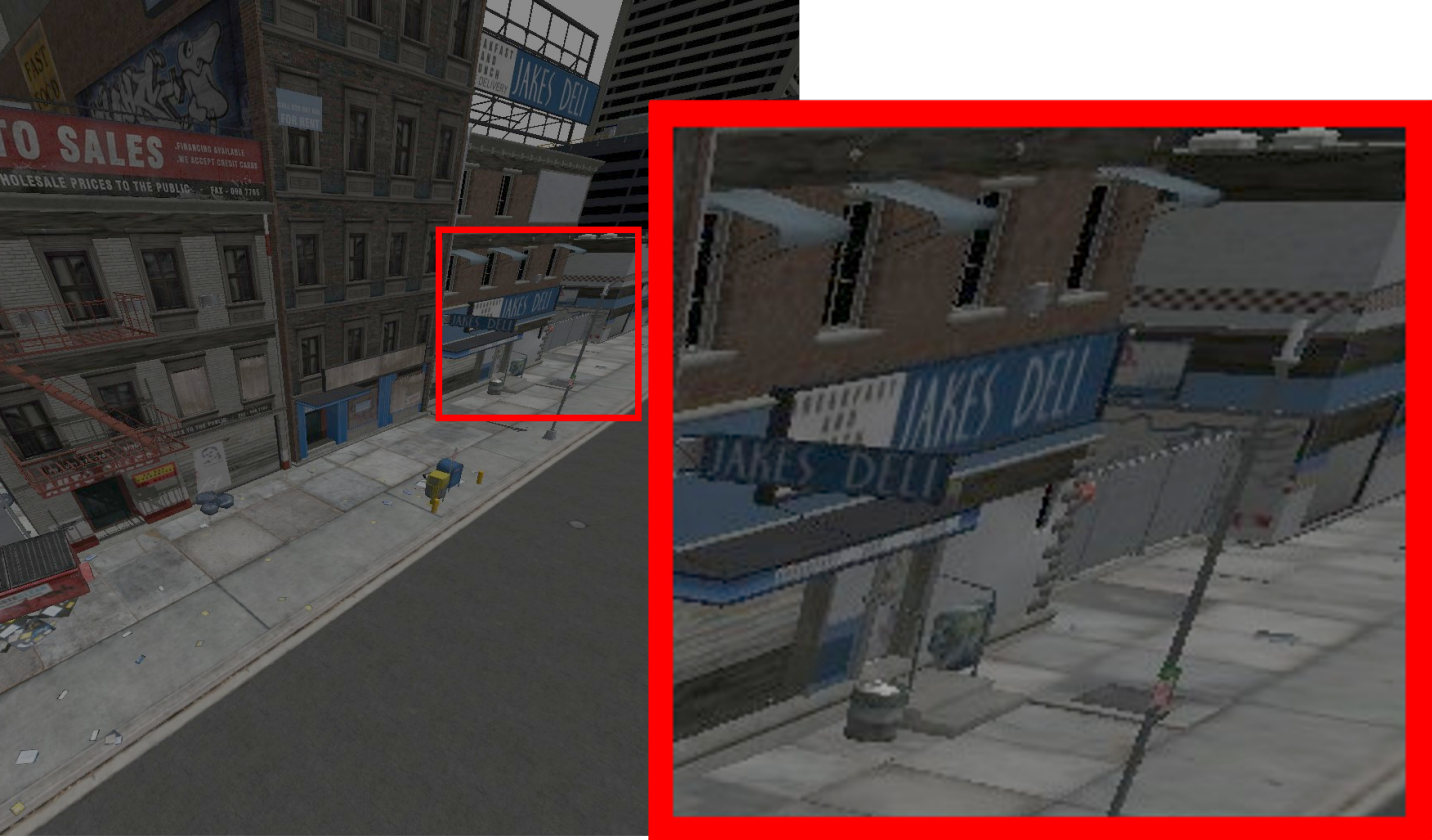

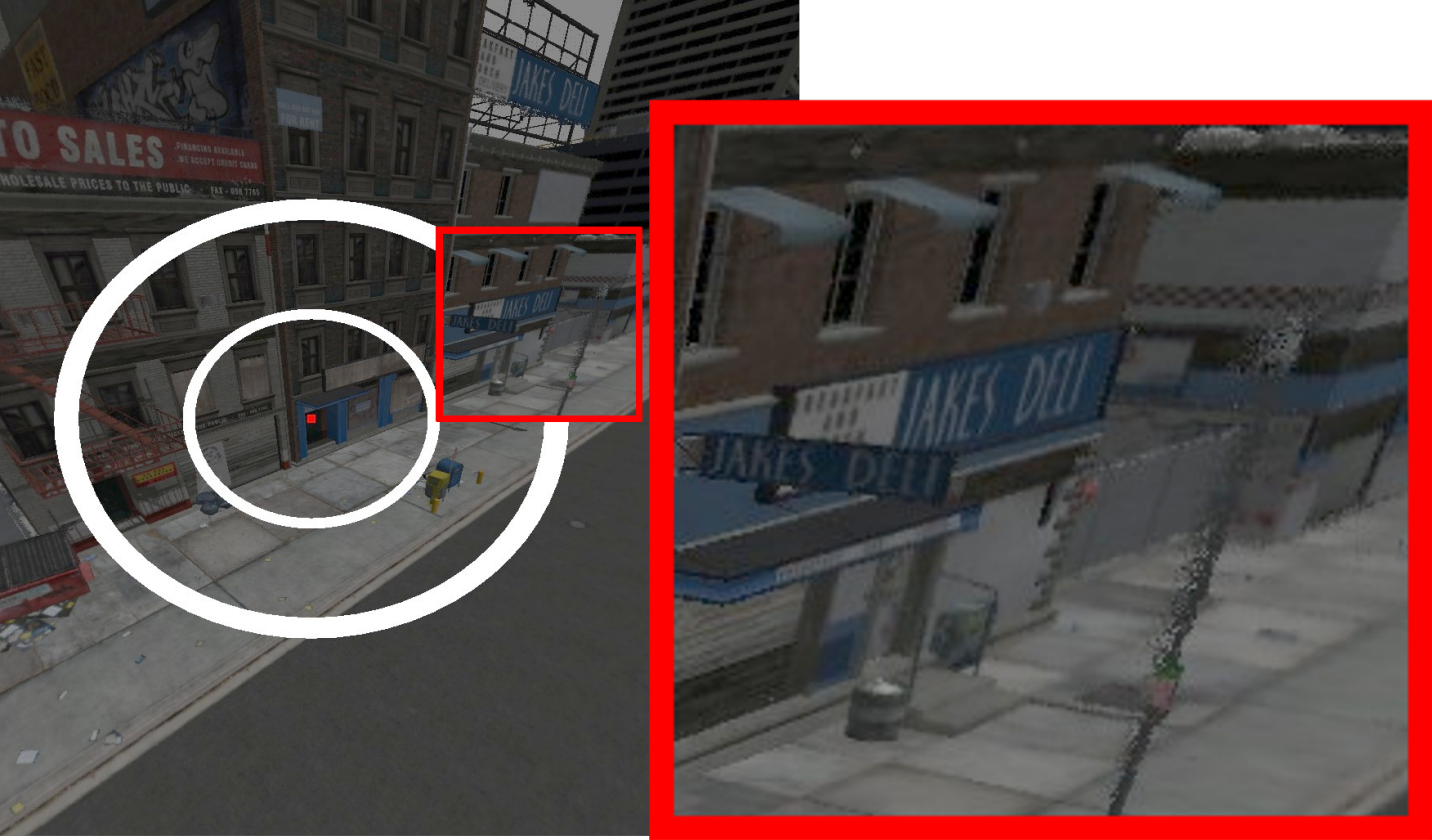
(a) to (d): Scenes used during our user study. (e), (f): Full ray tracing vs. foveated rendering. The white circles represent r_0_ and r_1_ in the foveal function

We carried out a user study to measure the visual quality of our rendering method. Our main goal was to answer our three research questions defined more clearly in [ 19 ]: 1. How well can users differentiate between foveated and non-foveated rendering? 2. How do varying foveal region configurations influence the subjective quality perception? 3. How do varying fixation modes affect the subjective quality perception? Each participant in our study was shown 96 trials resulting from a 4x4x3 full factorial within-subject design. Each of the trials consisted of the display of the fly-through (8 seconds) and a varying amount of time for the quality rating after each sequence. 15 subjects participated in our user study (10 male, 5 female). They were aged between 26 and 51 (M = 33, S D = 7.24) and all of them had an academic background. There was no compensation for participating in the experiment. The considered factors and the according levels were: 1. Four scenes {Sponza, TunnelGeom, TunnelMaps, Rungholt} (see Figure 2) 2. Four FRCs {small (5° ; 10° ; 0.01), medium (10° ; 20° ; 0.05), large (15° ; 30° ; 0.1), full (∞ ∞ 1)} 3. Three fixation types {fixed, moving, free}. Trials were shown to the participants in a randomized order, with each condition being presented twice, resulting in 4x4x3x2 = 96 trials.

 The main idea behind varying the fixation types was to find potential visual tunneling effects that had an influence on the outcome of the user study. In the fixed focus mode, a static fixation cross was displayed at the center of the screen. This had to be focused by the user for the entire trial. The moving target, on the other hand, consisted in a green, moving sphere. The position of this sphere was determined by paths that were generated randomly across the image area. For each individual path, the velocity of the fixation target was static (between 11 and 17 degrees per second).

To minimize learning effects, the utilized paths were varied in all trials except for repetitions. Identical combinations of all variables including the fixation paths were presented to all test subject, but in a randomized order. For both fixation modes, the foveal region was not controlled by the user, but centered around the fixation target. The additional free focus mode enabled the users to freely adjust the foveal region’s position with their eye movement. In this case, there is no reference for the desired PoR as in the other fixation modes. Nonetheless, we analyze the tracking data from the free focus mode in conjunction with given quality ratings and our measured tracking precision, giving additional hints about tracking precision and eccentricity-dependent quality perception.

Quality had to be rated by giving a level of agreement for two statements: "The shown sequence was free of visual artifacts." and "I was confident of my answer." Rating was done using a 7-point Likert scale which ranged from strongly disagree (-3) to strongly agree (3).

The tracking data was determined and recorded at a rate of 60Hz during all trials, while rendering was performed at a static update rate of 75Hz on an Oculus Rift DK2 HMD. The tracking data was mainly recorded in order to compare it to the prescribed paths.

The analysis of variances (ANOVA) and additional t-tests that were performed on the data collected during our user study have suggested that it is not possible to make a reliable di erentiation between our optimized rendering approach and full ray tracing, as long as the FRC is chosen to be at least of size medium. There were no significant di erences in quality ratings given for FRCs of medium, large and full.

The significant main e ect we have found for fixation types can likely be attributed to effects reducing the perception of some visual artifacts. The moving target mode was rated best over all scenes with a mean of 0.99 and a standard deviation of 1.63, while the static and free fixation resulted in a mean of 0.43 for both and a standard deviation of 1.81 for static and 1.89 for free fixation. The user study and rendering algorithm are both described in more detail in [ 19 ], also providing details regarding the statistical significance of the presented results.

Our goal now is to analyze the tracking data and the users’ corresponding quality ratings for the presence of effects like visual tunneling further than in [ 19 ], extending [ 22 ]. Effects like this may affect quality ratings in certain ways unexpected from the raw data. In addition, we aim for giving a deeper insight into tracking quality by also providing information on the relation between a user’s gaze and the given quality ratings. All distances in our analysis are average values of the left and the right eye.

To ensure that the recorded data is valid, we first tried to determine the actual tracking precision. When using the tracking device, it was quite noticeable that the precision degraded towards outer image areas. This may also be one reason for the calibration process of the eye tracker’s SDK only employing a relatively small area around the image center. Our estimate for tracking precision is given by looking at the deviations of the recorded PoR from the fixation target’s current position. Our basic assumption is that the fixation accuracy, describing how well a user can fixate a target, is largely independent of the target’s position in the image. Based on this assumption, it would follow that worse fixation towards outer areas most likely results from tracking inaccuracies.

To estimate tracking precision, we sort the data into bins, where it is then averaged. These bins have a width of w = 0.1° and there is a total of n = ⌈max(F_p;t_(i))/w⌉ bins B_j_ = (F¯_j_; G¯_j_); 0 ≤ j < n, with Equation 1 and Equation 2

**Figure:**
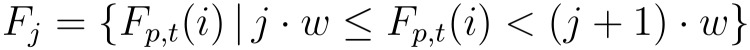


**Figure:**
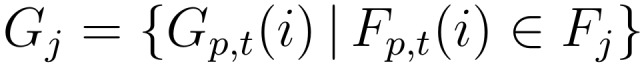


Here, F_p,t_(i) is the distance between the fixation target’s current position and the image center, while G_p,t_(i) represents the distance between the gaze and the fixation target in trial t at frame i for participant p. G¯ _j_, the average value for the according bin j, now provides an approximate tracking quality measure for the contained eccentricities, which would be [ j x w; ( j + 1) x w]. We analyze this data further by performing a linear regression, which is described in the results section below.

The average fixation accuracy of participants is then compared for all tested scenes. Eventually, we compare the measured fixation accuracies with quality ratings for individual scenes and try to explain the apparent effects. To support our findings regarding tracking precision, we analyze how quality ratings given by the users relate to average eccentricities of the points of regard in the free focus mode.

Adults can physically rotate the eye up to 50° horizontally, 42° up and 48° down around line of sight in the eye’s resting position [ 28 ]. However, it has to be noted that in practice, humans usually do not rotate the eye to the physiologically possible extent. After exceeding a certain angular deviation a human would highly likely start turning the head. This angular deviation is referred to as the comfortable viewing angle (CVA). It is considered to be ≈ 15° around the normal line of sight [ 29, p.17 ]. Thus, it is important to note that we did not account for fixation target eccentricities larger than the CVA in our tracking precision measures.

In our user study, head tracking was not implemented because it was necessary to present identical visual stimuli to all participants. This would not have been possible if users were able to freely look around. However, for fixation target positions further away from the image center than the CVA, users would most likely not just rely on eye movement to fixate a target, but instead incorporate head movement.

## Results

In this section we present the results of our analysis.

### Tracking Precision

**Figure 4:**
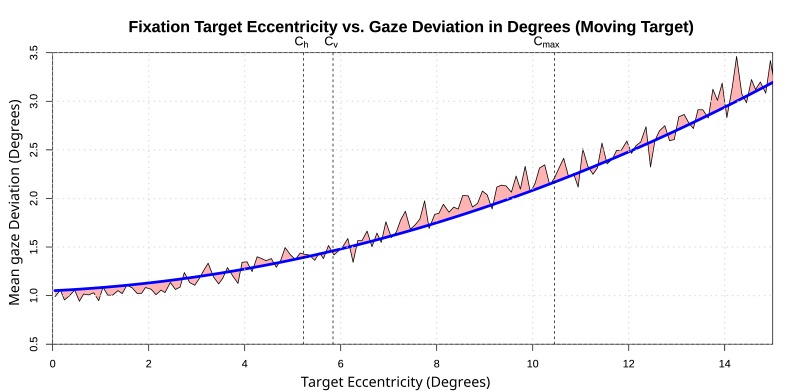
Tracking precision vs. fixation target’s distance to the image center. The area used for calibration by our tracking device is also denoted here by C_h_ (horizontal extent), C_v_ (vertical extent) and C_max_ (diagonal extent). The result of linear regression with a quadratic equation is represented by the blue line. The red area illustrates the residuals. Image adapted from [ 22 ].

To analyze in which way the tracking precision relates to the actual eccentricity of the PoR (which we assume to be identical to the fixation target position at this point), we perform a linear regression with Gˆ j = 0 + 1F j + 2F 2j. This results in a correlation of 0.989 with β = (1.05, 0.024, 0.008) and R^2^ = 0.978 with the constant (p≈ 0), linear (p < 0.01) and square (p≈ 0) terms being statistically significant. The quadratic prediction for gaze deviation is illustrated in Figure 4. The decreasing tracking precision for larger eccentricities becomes apparent from the regression result.

### Fixation Accuracy

**Figure 3:**
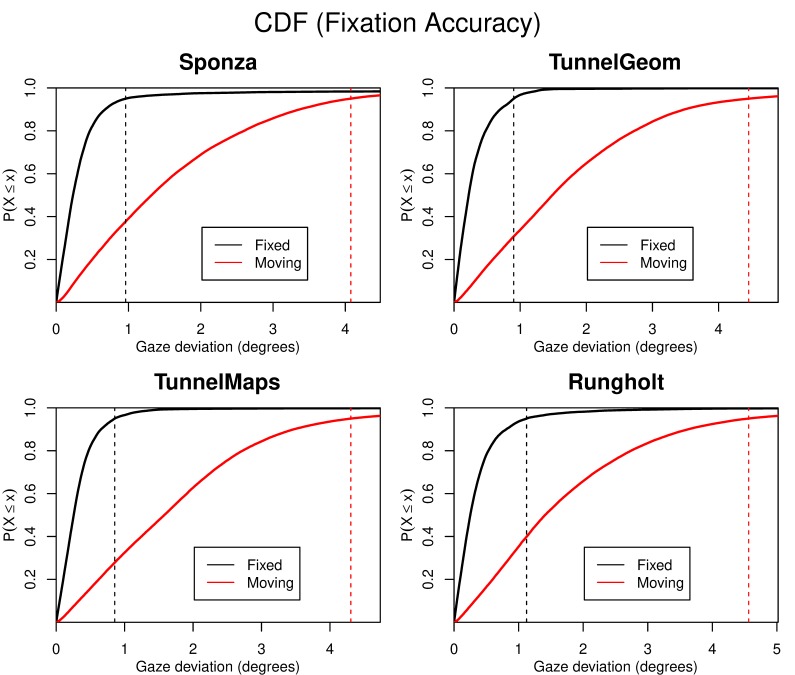
Cumulative distribution functions (CDFs) of the measured fixation accuracy for fixed and moving targets. The 95% quantiles of gaze deviations for each scene are illustrated with dotted lines. There are significant differences between the fixation accuracy for the fixed and the moving fixation targets. X is the actual gaze deviation.

**Figure 5:**
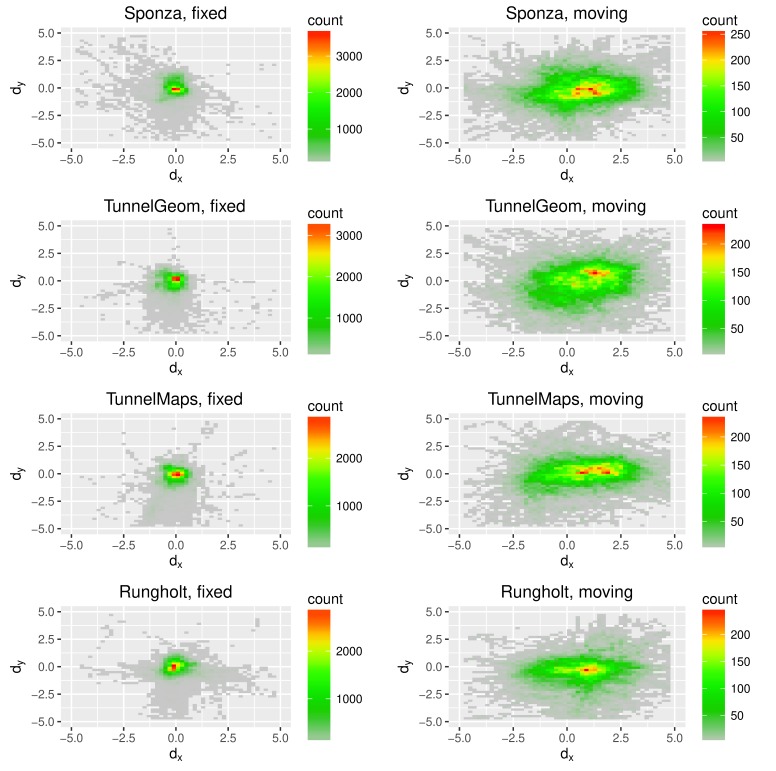
Gaze deviation for all individual scenes, fixed and moving targets.

Figure 3 shows the cumulative distribution functions (CDFs) for the fixed and the moving fixation target for all four scenes, with the horizontal axis representing the angular distance between the user’s gaze and the fixation target. It can be seen that there is a significant di erence between the fixation accuracy for the fixed target (below 1.1° ) and the moving target (approximately 4° to 4.5° for all scenes). In the discussion section, we explain the result that we would normally expect from this di erence in accuracy and put that into context with the users’ actual quality ratings. Figure 5 shows the distribution of gaze deviation for fixed and moving targets, respectively.

As indicated by the color range, it is also illustrated how often the users’ gaze has been found at the respective relative positions to the fixation target. The shift to the right for the gaze deviation can be explained by the utilized fixation paths not being equally distributed regarding the fixation target’s movement. We analyzed the paths, which revealed that the fixation target has moved left more often than right, which is one possible explanation for the slight shift of the PoR to the right on average. The di erence in the fixation accuracy between the moving target and the fixation cross is likely apparent because of the smooth pursuit eye movements (SPEM). Even though the speed of the moving object did not exceed the 100°/s were a decrease in accuracy is reported due to physiological constraints, the movement of the target was not predictable for the user. This naturally leads to a reduced SPEM precision. Moreover, precision is reduced due to the fact that the background lies at the same distance than the pursuit target. Thus other signals, e.g. by the vestibular system, cannot be used by the HVS to discriminate between target and background [ 28, p.229 ].

### Subjective Perceived Quality: Fixed and Moving Target

**Figure 6:**
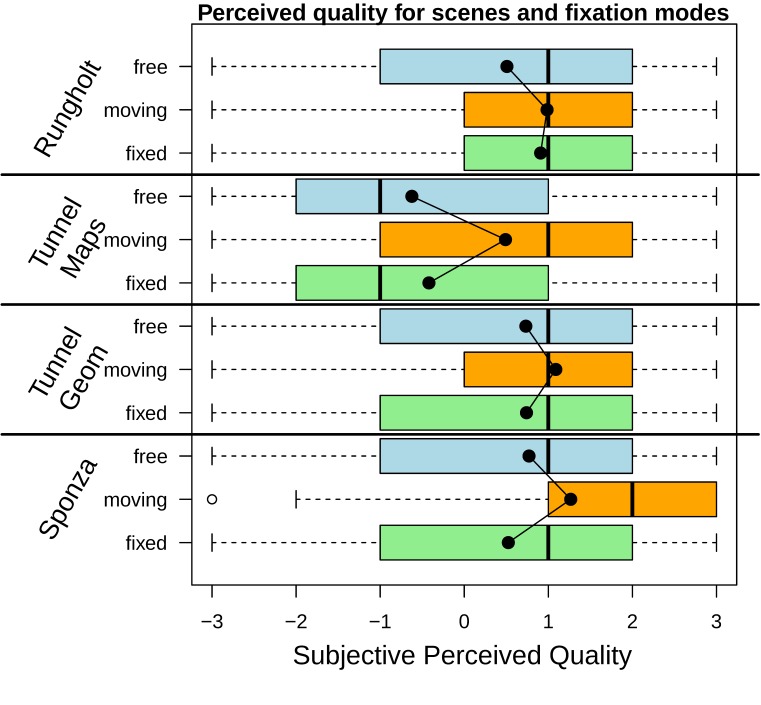
Quality for all combinations of scenes and fixation modes. Quality ratings were the highest for all scenes when the moving target fixation mode was selected, although the fixation accuracy was worse for the moving target than for the fixed target. The black dots inside the boxes represent the respective mean quality ratings.

**Figure 7:**
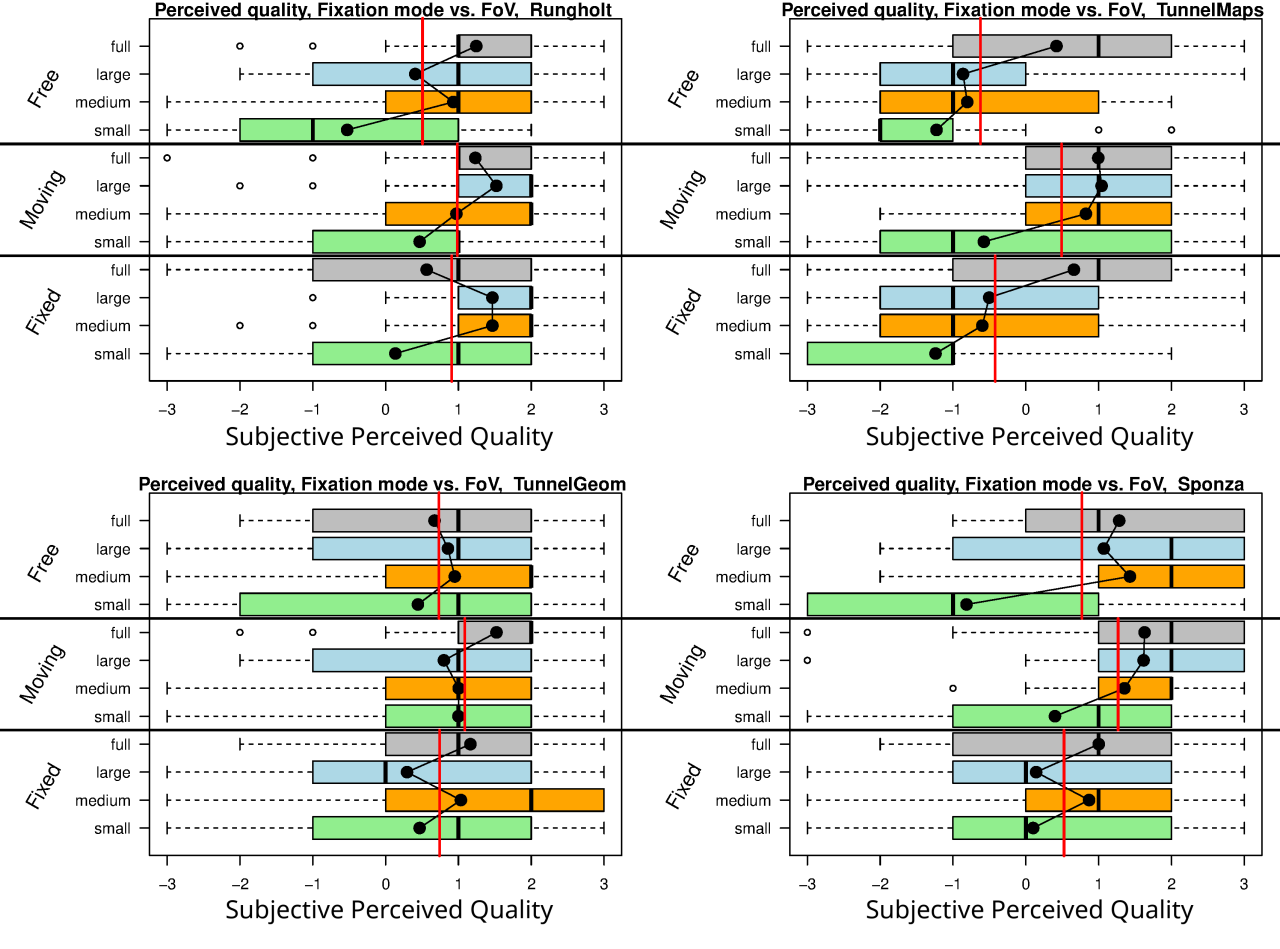
Quality ratings for fixation modes and Foveal Region Configurations, all scenes.

Figure 6. shows that the average quality for the moving target was rated better for all scenes on average. In order to shed some light on the influence of the actual rendering detail, Figure 7 illustrates the data for the individual scenes, each with all three fixation modes and all foveal region configurations up to full rendering. The red lines show the means for each of the fixation modes, exhibiting that the aforementioned effect is present in all tested scenes. It also becomes apparent that the increase in rendering detail between the medium and the large FRC did not result in a consistent improvement of subjective perceived quality. For the moving fixation target, di erences from a medium FRC up to full rendering are mostly negligible. Interestingly, in some cases a larger FRC even results in lower subjective perceived quality. We try to explain the given quality ratings in the discussion section, as they contradict intuition at first.

### Subjective Perceived Quality: Free Focus

**Figure 8:**
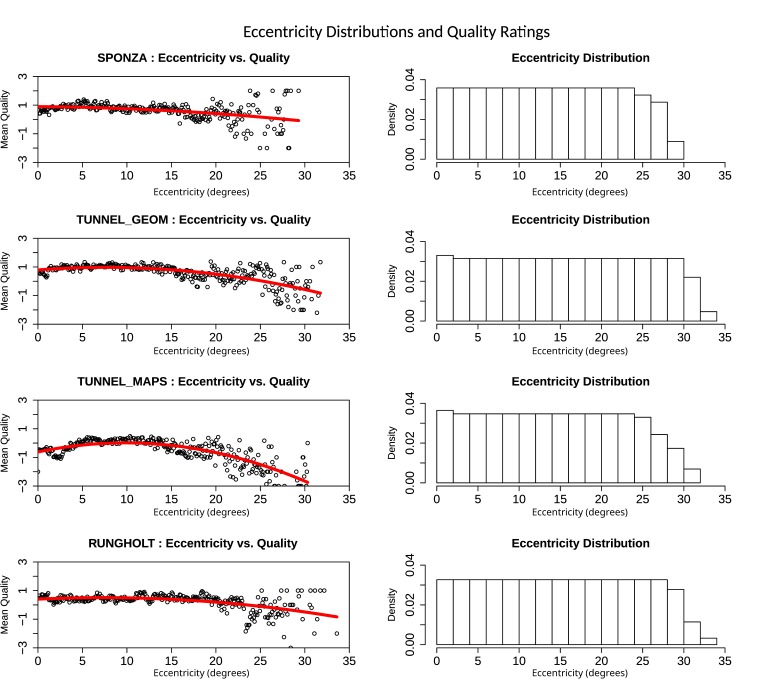
Eccentricity-dependent mean quality measurements and eccentricity distributions for all scenes for free focus mode. It is clearly visible that subjective perceived quality degrades with increasing eccentricities. Eccentricity distributions are similar and almost uniform for all scenes.

In the free focus mode, users were allowed to move their eyes freely instead of having to follow a prescribed path. As we have shown above, tracking quality seemingly degrades with increasing eccentricities. To prove that this apparent degradation does not only come from fixations, saccades and other disturbances not being filtered from the raw data, we take a look at the eccentricity-dependent quality ratings in the free focus mode. Figure 8 shows illustrations of the according data (eccentricity and quality ratings) for all scenes. The left column contains scatter plots for each scene. The horizontal axis represents the eccentricity, while the vertical axis represents the mean quality per bin, which has been computed for bins of size w = 0.1° . For the binning process, each recorded frame from all trials of a scene was analyzed for the tracked eccentricity, which was then used to account for the quality rating in the according bin. Another possible approach would be to average eccentricities for all individual trials and bin the data based on that. In addition to the mean quality for each of the bins, we performed linear regressions with quadratic equations, which are contained in each of the plots as a red curve. The right column shows eccentricity distributions for each scene, i.e., it gives an idea about how far away from the image center the user inspected the shown scenes. Di erences between scenes turn out to be mostly minuscule or at least too small to draw any further conclusions. See the discussion section for further remarks regarding these illustrations.

## Discussion

As shown above, the precision of the utilized eye tracking device drops with increasing eccentricities. Our solution at this point has been to limit the area accounted for in our considerations to only include fixation targets up to the CVA. Still, this issue could also be approached di erently. One possibility would be to take a deeper look into the calibration step of the eye tracking device. As Figure 4 illustrates, the tracking precision decreases smoothly with increasing eccentricities. It may be worthwhile to analyze di erent calibration procedures for their e ect on tracking precision. Also, our most recent tests have shown other devices to be possibly more capable of capturing accurate gaze data over a larger area. New generation eye trackers will likely improve on accuracy for greater angles. The result of our tracking precision analysis should not be interpreted as a direct measure for tracking precision, even though it seems to be quite accurate. Latency-based deviations, saccades and other possible disturbances have not been filtered from the data. The actual behaviour of the measured gaze deviation however yields a good estimate of the eccentricity-dependent precision fallo .

The lower fixation accuracy that we found for in moving target mode implies that the users PoR was often located within the border area of the foveal region for the small FRC. This exhibits reconstructed (and thus lower-quality) parts of the scene to the user in his/her central vision. Causes for the low accuracy that we measured are tracking latency and possible unpredictabilities of the target’s movement as well as tracking precision itself.

For the subjective perceived quality ratings, we have found that an increase in rendering detail did not always result in improved quality ratings. One possible cause for this is the reprojection method hiding visual artifacts by effectively putting a low-pass filter over them, as even full rendering – as all rendering methods – still is a subsampling of the rendered scene, just with a finer and more regular pixel grid. Thus, it may also contain visual artifacts. A more detailed explanation of blur effects that occur when using reprojection methods can be found in (?). Also, when rating the subjective perceived quality for the fixed targets on the one hand and the moving targets on the other hand, intuition may suggest a worse outcome for the latter because of the larger gaze deviation illustrated in Figure 3 and Figure 5. This assumption is mainly based on the fact that the visibility of image regions rendered at a lower quality is increased when the gaze deviation from the fixation target is higher. However, contrary to this, Figure 6 and Figure 7 reveal the quality ratings for moving target fixation to be better in all tested scenes. We interpret the consistent di erences in subjective perceived quality between fixation modes and their counterintuitive nature when taking tracking precision and temporal effects into account as evidence for the possible presence of visual tunneling effects. This means that visual artifacts that appear in our rendering system are effectively filtered by human perception, which makes them largely imperceptible. Also, there often was a clear tendency towards negative ratings for the small FRC. One possibility to overcome this issue is to enlarge the foveal region proportionally to the occuring eye movement. However, this approach poses a significant challenge. The achieved frame rate is already considered to be critical when it comes to head-mounted displays in general, and it becomes even more critical when incorporating (potentially rapid) eye movements. Contrariwise, increasing the rendering quality results in a performance hit, making it even more di cult to achieve the necessary refresh rate. Thus, this approach is only viable in an environment where enough computational resources are available. This may raise the question why these computational resources should not be initially put into rendering a larger FRC, but it has to be kept in mind that visualization is often only one part of an application and resources also need to be available for other components such as physics, interaction or AI, for example in computer games.

We have also shown results of the subjective perceived quality in the free focus mode (cf. Figure 8). As mentioned above, it becomes clear that subjective perceived quality in this mode degrades with increasing eccentricities. Besides a degradation on average, quality measurements also become rather unpredictable in areas further away from the image center. This may imply that the visual effects that occur through mismatches between the actual PoR and the measured PoR do not have the same e ect for all users. We suggest that this can be attributed mainly to the FRCs, as a large foveal region still presents the most important parts of the image at full detail to the user, while smaller FRCs tend to miss the user’s central vision completely due to tracking inaccuracies.

One of the challenges that are yet unsolved is the issue of HMDs getting out of place in the process of a user study, or, more generally, during the execution of an application or specific task. Even slight movements of the HMD may lead to an eye tracker’s calibration becoming invalid. However, asking the user to repeat the calibration step each time the HMD has moved too much is not a viable option. In opposition to an explicit calibration procedure, having a calibration that is embedded into the task at hand would make HMDs with eye trackers more practical for everyday applications.

## Conclusion

In this paper we have analyzed the data recorded by an eye tracking device during the evaluation of our foveated rendering method. We described our evaluation setup as well as the rendering method itself. Tracking precision has been analyzed regarding its angular dependencies, revealing a clear drop of tracking quality for higher eccentricities. Accordingly, quality ratings for free focus mode also show a clear drop towards larger eccentricities. Properties of tracking devices such as this have to be accounted for when implementing foveated rendering methods, as the point of regard is a crucial measurement in such setups. Having measured these inaccuracies of the tracking device, it becomes clear that applications that rely on methods from this field have to adjust the specific parameterizations for the given circumstances.

We have analyzed the ability of users to focus static and moving fixation targets. While we found the PoR being scattered over larger areas for the moving target mode, the results seemed to contradict the intuitive assumption that worse fixations should result in worse quality ratings. The mean quality ratings were best for the moving target mode in all scenes, even though the match between the measured PoR and the actually focused PoR was worse than for the static fixation mode. Even though this may lead to subsampling and reprojection artifacts being exposed to the user, the ratings were still better, which we attribute to the potential presence of visual tunneling effects that are induced by the mental load of the task that has to be carried out, although the task has just been to follow a moving point. effectively, this reduces the user’s field of view.

Thus, there are circumstances which make it possible to reduce visual quality. This is the case in games, where events can be triggered that produce a change in the visuals, or taskdriven environments, where task or navigation complexity may lead to high mental workloads. Moreover, certain events may allow for deriving a hint which part of the scene attracts attention. Thus, visual quality can be reduced even further (Selective rendering). However, attentional models and gaze predictions are far from accurate [ 5 ] However, more recently the flicker observer e ect and the higher temporal resolution for peripheral vision has successfully been used to direct the user’s gaze directly [ 30 ].

Future research in the area of foveated rendering may analyze further how optimal foveal region configurations can be determined and how the point of regard can be optimally placed even with imprecise tracking. Also, it may be possible to exploit visual tunneling effects directly to improve performance or, alternatively, visual quality for central vision. In addition, it may be worth looking into the comparative behavior of tracking devices with di erent update rates for analyses such as the one we have presented here.

## Acknowledgements

We would like to thank NVIDIA for providing us with two Quadro K6000 graphics cards for the user study, the Intel Visual Computing Institute, the European Union (EU) for the co-funding as part of the Dreamspace project, the German Federal Ministry for Economic A airs and Energy (BMWi) for funding the MATEDIS ZIM project (grant no KF2644109) and the Federal Ministry of Education and Research (BMBF) for funding the project OLIVE (grant no 13N13161).

## Conflict of Interest

The authors declare that there is no conflict of interest regarding the publication of this paper.
